# Diagnostic accuracy of resting left ventricular akinesia/hypokinesia in predicting abnormal coronary angiography

**DOI:** 10.1186/s12872-016-0312-5

**Published:** 2016-06-13

**Authors:** Mohamed Faisal Lutfi

**Affiliations:** Department of Physiology, Faculty of Medicine and Health Sciences, Al-Neelain University, Mailbox: 12702, Khartoum, 11121 Sudan

**Keywords:** Akinesia/hypokinesia, Coronary artery disease, Coronary angiography, Ejection fraction

## Abstract

**Background:**

Although several reports demonstrate the efficacy of stress echocardiography in diagnosing coronary artery disease, comparable studies on the competence of the same imaging technique at rest are limited. This study aimed to evaluate whether ventricular akinesia/hypokinesia and left ventricular ejection fraction (LVEF) < 55 % at rest are useful in predicting abnormal coronary angiography.

**Methods:**

This study prospectively enrolled 100 diagnostic coronary catheterization candidates. Any routine echocardiography that the candidates had undergone before diagnostic coronary catheterization was reviewed. Patients were subclassified according to the presence and location of ventricular akinesia/hypokinesia, LVEF, and the results of diagnostic coronary catheterization. LVEF < 55 % was considered below the normal physiological limit. Abnormal coronary angiography was defined as narrowing of half or more of the caliber of at least one major coronary artery.

**Results:**

Abnormal coronary angiography was significantly associated with akinesia/hypokinesia (OR = 4.85, *P* = 0.002) and LVEF < 55 % (OR = 5.75, *P* = 0.001). Screening of akinesia/hypokinesia and LVEF < 55 % as diagnostic tools for abnormal coronary angiography achieved comparable sensitivities (87.2 % vs. 88.9 %), specificities (41.5 vs. 41.8), and diagnostic accuracies (41.5 vs. 41.8). Left ventricular anterior wall akinesia/hypokinesia achieved a higher diagnostic odds ratio (9.7), sensitivity (95 %), and negative predictive value (96.4 %) compared with other types of akinesia/hypokinesia.

**Conclusion:**

The overall diagnostic accuracy of akinesia/hypokinesia and LVEF < 55 % to predict abnormal coronary angiography was poor, probably owing to significant influences of macro- as well as micro-vascular ischemia on left ventricular function.

## Background

Myocardial ischemia is known to depress cardiac contractility [1]. Reduction of left ventricular wall motion (LVWM) and/or ejection fraction (LVEF) usually indicates myocardial ischemia unless proven otherwise [2]. Depressed left ventricular function (LVF) is likely, whether myocardial ischemia is owing to compromised function of large epicardial vessels [3] or the microcirculation of the heart [4, 5]. The presence of clinical features of myocardial ischemia usually indicates coronary artery disease (CAD) [6]. In such patients, failure of the coronary angiography to demonstrate apparent, or induced, narrowing of the large coronary vessels redirects the possible diagnosis towards microvascular ischemia of the myocardium. Cardiac syndrome X (CSX) is commonly used to describe the triad of: typical cardiac chest pain, significant changes in one or more of the cardiac stress test(s), and normal angiography of the epicardial coronary vessels [7, 8].

Cardiac stress tests evaluate the possible ischemic electrocardiography (ECG) changes, depressed LVWM or LVEF while heart activity is increased by exercise or other inotropic factors [9, 10]. Although there are several reports on the efficiency of stress echocardiography in the diagnosis of CAD [11, 12], comparable studies on the competence of the same technique at rest are limited [13]. Clinicians in developing countries are usually forced to use resting ECG and echocardiography for the diagnosis and risk stratification of CAD patients, owing to a lack of facilities and poor financial resources. In Sudan, selection of candidates for diagnostic coronary catheterization (DCC) is partly dependent on depressed LVWM and/or LVEF on resting echocardiography. The present study aimed to evaluate ventricular akinesia/hypokinesia and LVEF < 55 % derived from resting echocardiography as screening tests for the detection of abnormal coronary angiography (ACA). This is the first study to evaluate these two measures as screening tools for ACA in Sudan and probably worldwide.

## Methods

This study received clearance from the Ethics Review Committee of the Faculty of Medicine, Khartoum University, Sudan. Written informed consent was provided by each volunteer before being enrolled in the study.

This study prospectively enrolled 100 diagnostic coronary catheterization (DCC) candidates in Al-Shaab Cardiac Center, Khartoum, Sudan. Following evaluation of sociodemographic characteristics, past medical history, and clinical examination of each patient, results of routine echocardiography conducted before DCC were reviewed. Patients were subclassified according to the presence and location of ventricular akinesia/hypokinesia, LVEF, and the results of DCC. The endocardial and epicardial motion, as well as thickening of each segment of the LV, were assessed to assign a wall motion score index (WMSI). Normal wall thickening (WMSI = 1) was considered indicative of normokinesia, while decreased (WMSI = 2) and absent (WMSI = 3) wall thickening were considered indicative of hypokinesia and akinesia respectively. Left ventricular anterior (LVAW), septal (LVSW), inferior (LVIW), and lateral (LVLW) walls were assessed for the presence of akinesia/hypokinesia, irrespective of which particular part of the wall was affected. According to the 16-LV segmentation model, LVSW included the basal anteroseptal, basal inferoseptal, mid-anteroseptal, mid-inferoseptal, and apical parts of the interventricular septum; LVAW included the basal, mid, or apical parts of the anterior LV wall; LVIW included the basal, mid, or apical parts of the inferior LV wall; and LVLW included the basal anterolateral, basal inferolateral, mid-anterolateral, mid-inferolateral, and apical parts of the lateral wall. LVEF < 55 % was considered to be below the normal physiological limit [14]. Patients with narrowing of half or more of the caliber of one or more of the major coronary arteries were considered to have ACA [15]. The following formula was used to estimate body mass index (BMI):

$$ \mathrm{B}\mathrm{M}\mathrm{I}\ \left(\mathrm{K}\mathrm{g}/\mathrm{m}\hat{\mkern6mu} 2\right) = \mathrm{weight}\ \left(\mathrm{kg}\right)/\Big(\mathrm{height}\ \left(\mathrm{m}\hat{\mkern6mu} 2\right). $$

Statistical analysis was performed using OpenEpi software, version 2.3, and Statistical Package for the Social Sciences (SPSS) for Windows, version 16.0 (SPSS Inc., Chicago, IL, USA). Studied variables were described as the mean ± standard deviation. Proportions of the studied groups were expressed as percentages (%) and 95 % confidence intervals (CI). The unpaired *t*-test was used to evaluate differences in the means of the studied variables between patients with normal coronary angiogram (NCA) and ACA. The binary logestic regression was used to evaluate the association between ACA and akinesia/hypokinesia as well as LVEF < 55 %. Sensitivity, specificity, positive predictive value (PPV), negative predictive value (NPV), diagnostic accuracy (DA), likelihood ratio of a positive test (LRP), likelihood ratio of a negative test (LRN), and diagnostic odds ratio (DOR) were calculated to evaluate akinesia/hypokinesia and LVEF < 55 % as screening tests for ACA. *P* < 0.05 was considered significant.

## Results

**Fig. 1:**
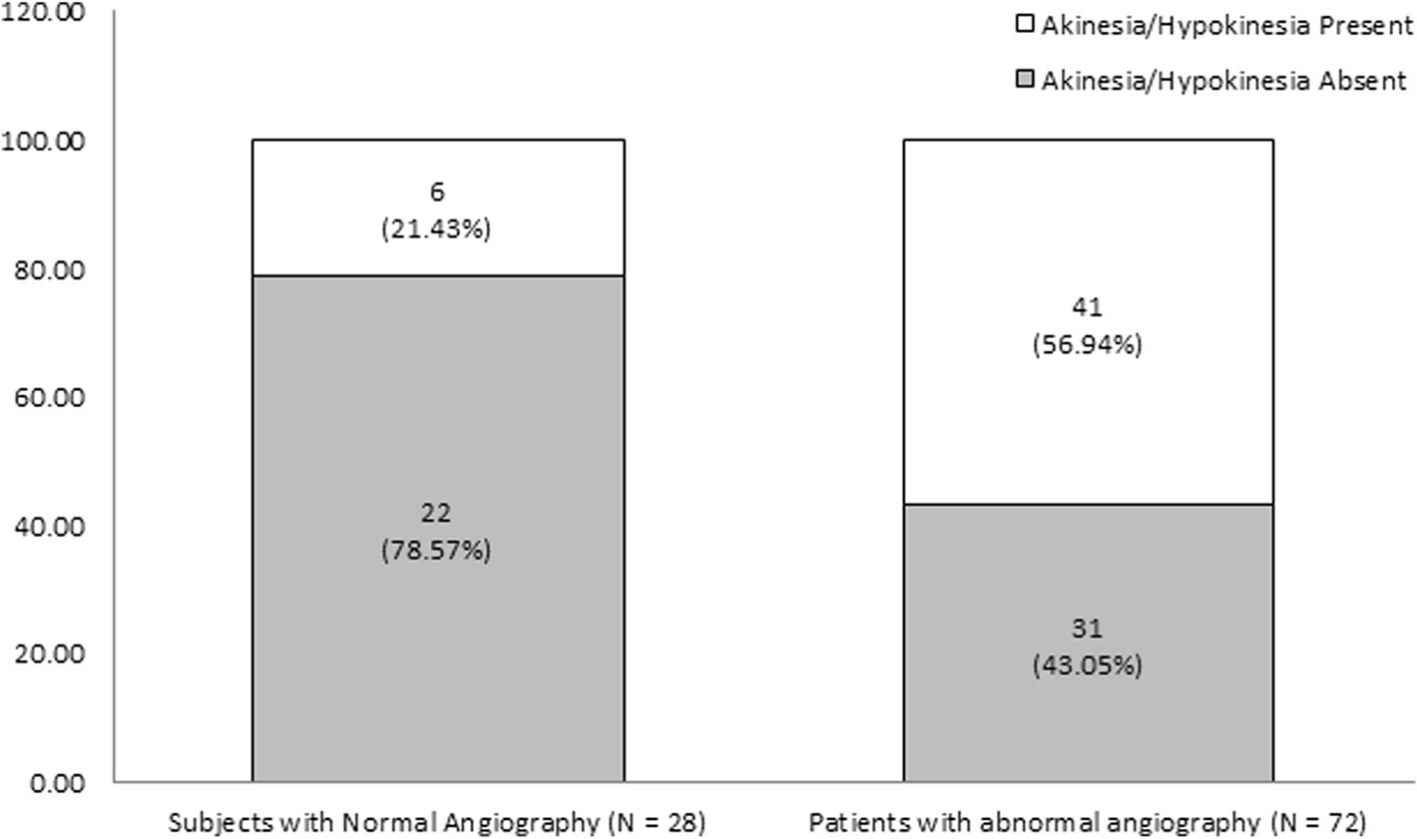
Distribution of akinesia/hypokinesia among the studied groups

**Fig. 2:**
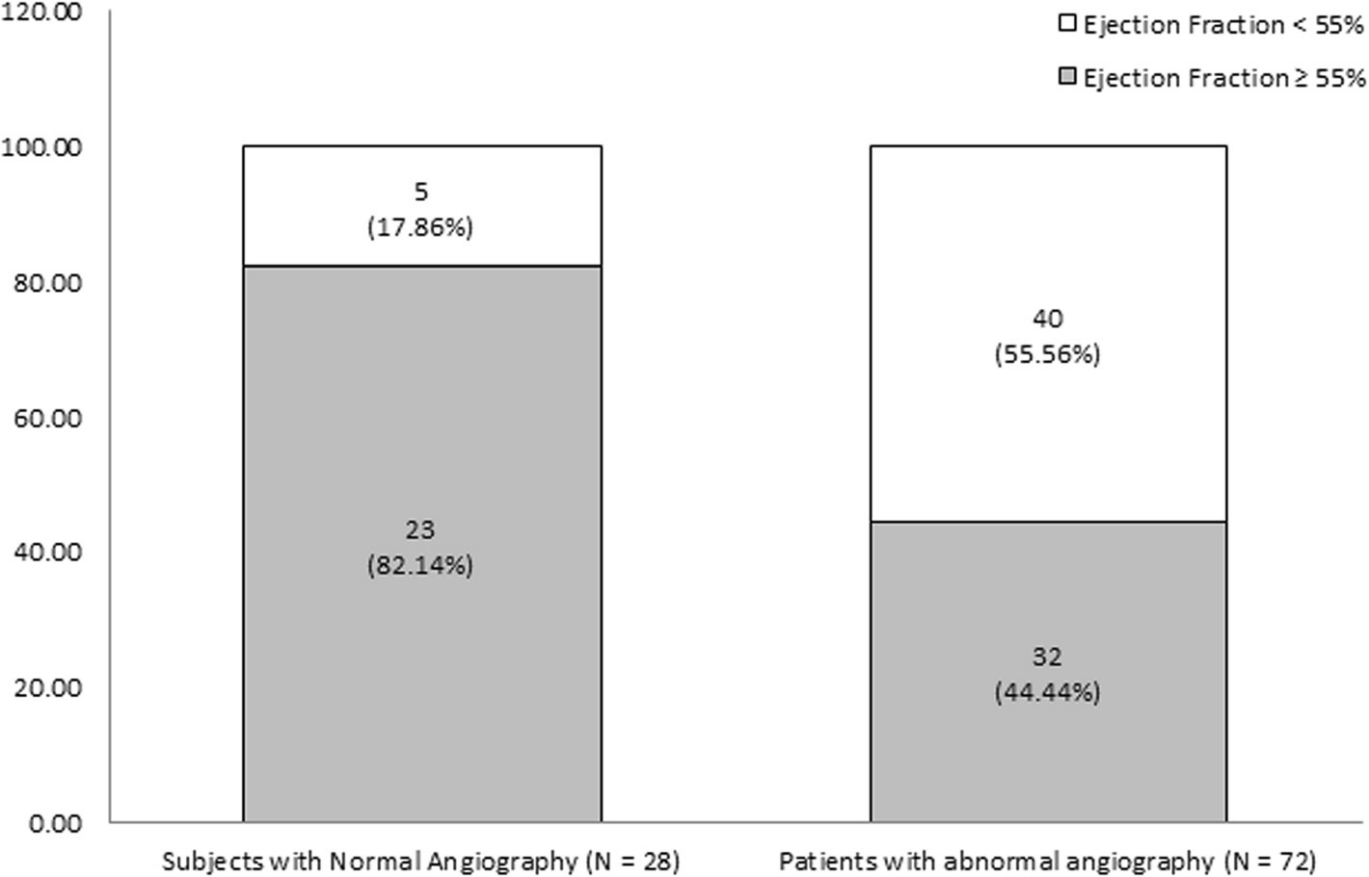
Distribution of left ventricular ejection fraction values (< or ≥ 55 %) among the studied groups

Coronary angiography confirmed CAD in 72 of 100 patients subjected to DCC (72 %, 95 % CI 62.5–79.9 %). Eight of the subjects with NCA were male (28.6 %, 95 % CI 15.3–47.1 %), and 53 of the subjects with ACA were male (73.6 %, 95 % CI 62.4–82.4 %). Characteristics of the studied groups are given in Table 1. Patients with ACA were significantly older (59.58 ± 9.80 years) and had a significantly lower BMI (25.70 ± 3.97 kg/m^2^) compared with those with NCA (49.11 ± 8.16 years, 29.58 ± 4.96 kg/m^2^; *P* < 0.001). ACA was significantly associated with male sex (OR = 6.97, *P* < 0.001), diabetes mellitus (OR = 3.89, *P* = 0.013), akinesia/hypokinesia (OR = 4.85, *P* = 0.002; Fig. 1), and LVEF < 55 % (OR = 5.75, *P* = 0.001; Fig. 2). Evaluation of akinesia/hypokinesia and LVEF < 55 % as screening tests for ACA revealed highly comparable conditional ratios (Table 2). The DORs and sensitivities of akinesia/hypokinesia and LVEF < 55 % as screening tools for diagnosis of CAD were relatively higher than the other conditional ratios (Table 2). LVAW akinesia/hypokinesia achieved the highest DOR (mean 9.7 %; range, 1.2–76.2 %), sensitivity (95 %), and NPV (96.4 %) compared with the other locations (Table 3). Specificities and PPVs were equally poor in all types of akinesia/hypokinesia (Table 3).

**Table 1:** Characteristics of the studied groups

	Subjects with normal angiography	Subjects with abnormal angiography	P
	*N* = 28	*N* = 72	
	M (SD)	M (SD)	
Age (Years)	49.11 (8.16)	59.58 (9.80)	<0.001
BMI (kg/m2)	29.58 (4.96)	25.70 (3.97)	<0.001
SBP (mmHg)	129.25 (18.83)	129.49 (24.87)	0.963
DBP (mmHg)	77.64 (11.42)	77.94 (13.06)	0.918
LVEF (%)	59.50 (9.77)	52.10 (12.69)	0.003

*M* mean, *SD* standard deviation, *BMI* body mass index, *SBP* systolic blood pressure, *DBP* diastolic blood pressure, *LVEF* left ventricular ejection fraction

**Table 2:** Evaluation of akinesia/hypokinesia and left ventricular ejection fraction < 55 % as screening tests for coronary artery disease

	Akinesia/Hypokinesia	EF < 55 %
	Estimate (95 % CI)	Estimate (95 % CI)
Sensitivity (%)	87.2 (74.8–94.0)	88.9 (76.5–95.2)
Specificity (%)	41.5 (29.2–54.9)	41.8 (29.7–55.0)
PPV (%)	56.9 (45.4–67.7)	55.6 (44.1–66.5)
NPV (%)	78.6 (60.5–89.8)	82.1 (64.4–92.1)
DA (%)	63.0 (53.2–71.8)	63.6 (53.8–72.4)
LRP	1.5 (1.4–1.6)	1.5 (1.4–1.6)
LRN	0.3 (0.2–0.5)	0.3 (0.2–0.4)
DOR	4.8 (1.8–13.4)	5.8 (2.0–16.8)

*EF* ejection fraction, *CI* confidence intervals, *PPV* positive predictive value, *NPV* negative predictive value, *DA* diagnostic accuracy, *LRP* likelihood ratio of a positive test, *LRN* likelihood ratio of a negative test, *DOR* diagnostic odds ratio

**Table 3:** Evaluation of different locations of left ventricular akinesia/hypokinesia in the screening of coronary artery disease

	Anterior wall akinesia/Hypokinesia estimate (95 % CI)	Septal akinesia/Hypokinesia estimate (95 % CI)	Inferior wall akinesia/Hypokinesia estimate (95 % CI)
Sensitivity (%)	95.0 (76.4–99.1)	89.3 (72.8–96.3)	87.0 (67.9–95.5)
Specificity (%)	33.8 (24.4–44.6)	34.7 (24.8–46.2)	32.5 (23.1–43.5)
PPV (%)	26.4 (17.6–37.6)	34.7 (24.8–46.2)	27.8 (18.8–39.1)
NPV (%)	96.4 (82.3–99.4)	89.3 (72.8–96.3)	89.3 (72.8–96.3)
DA (%)	46.0 (36.6–55.7)	50.0 (40.4–59.6)	45.0 (35.6–54.8)
LRP	1.4 (1.4–1.5)	1.4 (1.3–1.4)	1.3 (1.2–1.4)
LRN	0.1 (0.01–1.2)	0.3 (0.1–0.7)	0.4 (0.2–0.9)
DOR	9.7 (1.2–76.2)	4.4 (1.2–16.1)	3.2 (0.9–11.8)

*CI* confidence intervals, *PPV* positive predictive value, *NPV* negative predictive value, *DA* diagnostic accuracy, *LRP* likelihood ratio of a positive test, *LRN* likelihood ratio of a negative test, *DOR* diagnostic odds ratio

## Discussion

Presence of LV akinesia/hypokinesia and/or LVEF < 55 % significantly increased the odds of having ACA in patients undergoing DCC. Currently, LVWM and LVEF are important indicators of LVF, and are commonly used to assess severity and prognosis of CAD [2]. In one study, echocardiographic estimates of LVEF were validated using ventriculography measurements of LVEF for comparison; LVWM-derived estimates of ejection fraction faithfully reflected LVEF measured by the standard reference method [16]. Another study by Squeri et al. [3] demonstrated the intimate relationship between LVEF and severity of CAD. They assessed stress-induced changes in LVEF (∆LVEF) in four groups: controls, and patients with single-, two-, and three-vessel disease. The results showed that mean ΔLVEF was negative in patients with three-vessel or left main CAD, indicating that decreased heart contractility followed stress-induced myocardial ischemia [3]. In addition, the ΔLVEF was significantly lower in all other angiographic groups compared with the controls [3]. These previous findings thus reflect the efficiency of LVEF in evaluating the severity of CAD. The present study found that major impairment of LVWM and LVEF increased the odds of having ACA by about five to six times, which gives further support to the findings reported by Squeri et al. [3].

In the present study, the relatively higher sensitivity compared with specificity of akinesia/hypokinesia and LVEF < 55 % as screening tools for ACA suggests that these tests are more efficient in detecting, but not excluding, patients with ACA [17]. The higher NPV relative to PPV suggests a higher proportion of NCA subjects in those with normal LVF compared with the proportion of ACA patients in those with akinesia/hypokinesia and LVEF < 55 % [18]. PPV and NPV are influenced by the disease prevalence [19]; higher prevalence tends to result in increased PPV and decreased NPV [20]. The prevalence of CAD is relatively lower in Sudan compared with developed countries, and is therefore likely to have affected the PPV and NPV assessed in the present study [21]. Hence, LV akinesia/hypokinesia and LVEF < 55 % were also evaluated by LRP and LRN, which showed comparable findings to PPV and NPV [22, 23]. According to the LRP results, akinesia/hypokinesia and LVEF < 55 % was 1.5 times more likely in cases with ACA than those with NCA. The LRN values suggested that normal LVF was 0.3 times more likely in cases with ACA than those with NCA. The wide gap between sensitivity and specificity, as well as between PPV and NPV, indicates poor overall DA of akinesia/hypokinesia and LVEF < 55 % as screening tests for ACA. The variations in the conditional ratios were maintained in different locations of akinesia/hypokinesia when evaluated as screening tests for ACA. LVAW akinesia/hypokinesia achieved the highest DOR, sensitivity, and NPV, and equally poor specificity and PPV, compared with the other locations of akinesia/hypokinesia.

Although it is evident from the current results and previous reports that abnormal LVWM and LVEF are common in CAD patients, the presence of such findings cannot exclude microvascular ischemia. The coexistence of angina and ischemic ECG changes with uneventful DCC in patients with NCA in the current study is highly suggestive of CSX in this group [7, 8]. According to one study, when CSX patients are subjected to exercise, LV function is maintained as long as there is no ST segment shift; worsening of LV function is proportional to the degree of ST segment depression [5]. These findings are further supported by another study that showed decreased myocardial perfusion in 47 % and abnormal wall motion in 35 % of CSX patients following stress testing [4]. The same study concluded that microcirculatory dysfunction of the myocardium in some patients with CSX can result in concordant transient segmental LVWM abnormalities and impaired LVEF [4]. The findings of these previous studies on the pattern of changes in patients with macro- and micro-vascular ischemia explain the poor performance of akinesia/hypokinesia and LVEF < 55 % as screening tools for prediction of ACA.

The current study had some limitations. Although 100 DCC candidates were enrolled, a larger sample size would have enabled more definitive conclusions to be made regarding the diagnostic accuracy of resting LV akinesia/hypokinesia in predicting ACA. In addition, the lack of stress echocardiography testing in Sudan precluded evaluation of LV akinesia/hypokinesia in the studied patients during increased heart activity. Combined evaluation of DCC patients with resting and stress echocardiography tests in the future could offer scientific evidence as to which test has better DA in predicting ACA.

## Conclusion

Evaluation of akinesia/hypokinesia and LVEF < 55 % as screening tools for CAD suggests that these tests were more reliable for detection, but not exclusion, of patients with ACA. Overall DA of these measures to discriminate ACA from NCA was poor, probably owing to the comparable influence of macro- and micro-vascular ischemia on LVF.

## Abbreviations

∆LVEF, change in LVEF; ACA, abnormal coronary angiography; BMI, body mass index; CAD, coronary artery disease; CI, confidence intervals; CSX, cardiac syndrome X; DA, diagnostic accuracy; DBP, diastolic blood pressure; DCC, diagnostic coronary catheterization; DOR, diagnostic odds ratio; ECG, electrocardiography; LRN, likelihood ratio of a negative test; LRP, likelihood ratio of a positive test; LVAW, left ventricular anterior wall; LVEF, left ventricular ejection fraction; LVF, left ventricular function; LVIW, left ventricular inferior wall; LVPW, left ventricular posterior wall; LVSW, left ventricular septal wall; LVWM, left ventricular wall motion; M, mean; NCA, normal coronary angiography; NPV, negative predictive value; PPV, positive predictive value; SBP, systolic blood pressure; SD, standard deviation; SPSS, Statistical Package for the Social Sciences
